# Carbon-Bonded Alumina Filters Coated by Graphene Oxide for Water Treatment

**DOI:** 10.3390/ma13082006

**Published:** 2020-04-24

**Authors:** Ondřej Jankovský, Michal Lojka, Adéla Jiříčková, Christos G. Aneziris, Enrico Storti, David Sedmidubský

**Affiliations:** 1 Department of Inorganic Chemistry, Faculty of Chemical Technology, University of Chemistry and Technology, Technická 5, 166 28 Prague 6, Czech Republic; michal.lojka@vscht.cz (M.L.); Adela.Jirickova@vscht.cz (A.J.); sedmidub@vscht.cz (D.S.); 2 Institute of Ceramic, Glass and Construction Materials, Technische Universität Bergakademie Freiberg, Agricolastraße 17, 09599 Freiberg, Germany; aneziris@ikgb.tu-freiberg.de (C.G.A.); enrico.storti@ikgb.tu-freiberg.de (E.S.)

**Keywords:** graphene oxide, water treatment, ceramic foam filters, carbon-bonded alumina

## Abstract

The aim of this paper is to prepare nano-functionalized ceramic foam filters from carbon-bonded alumina. The carbon-bonded filters were produced via the Schwartzwalder process using a two-step approach. The prepared ceramic foam filters were further coated using graphene oxide. Graphene oxide was prepared by the modified Tour method. The C/O of the graphene oxide ratio was evaluated by XPS, EDS and elemental analysis (EA). The amount and type of individual oxygen functionalities were characterized by XPS and Raman spectroscopy. The microstructure was studied by TEM, and XRD was used to evaluate the interlayer distance. In the next step, filters were coated by graphene oxide using dip-coating. After drying, the prepared composite filters were used for the purification of the water containing lead, zinc and cadmium ions. The efficiency of the sorption was very high, suggesting the potential use of these materials for the treatment of wastewater from heavy metals.

## 1. Introduction

Ceramic foam filters (Al_2_O_3_–C, Al_2_O_3_, SiC–Al_2_O_3_, Y-doped ZrO_2_) are commonly used by foundries, in order to capture non-metallic inclusions from the metal melts [1]. Without filtration, such impurities can produce internal cracks, slivers or blisters in final rolled products. Furthermore, large clusters of inclusions significantly impact the mechanical properties [2]. Nowadays, the majority of the applied filters are open-cell ceramic foams produced via the replica process [3]. These filters can be further functionalized or modified due to their relatively high specific surface area. Carbon-bonded alumina foam filters are widely used for the steel filtration. Because of their structure and chemistry, these filters can be modified by graphene oxides or graphene [4].

The first preparation of graphite oxide (GO) was already reported in the nineteenth century [5], and several possible structures of GO have been reported [6,7,8,9,10,11]. Generally, graphite oxide is a lamellar compound, consisting of carbon layers with oxygen-containing functionalities (hydroxyls, epoxides, ketones, carboxyls etc.). Nevertheless, the exact structure of graphite oxides or graphene oxides is strongly influenced by the used starting material or oxidation process [12,13]. Let us note that that the graphene oxide contained less than 10 layers, while graphite oxide is a bulk material. The interlayer distance of GO is significantly increasing during the oxidation process (from 3.4 Å in graphite to 6–10 Å [14,15]). Highly oxidized graphite oxide can be simply exfoliated by sonication. 

The C/O ratio of GO is dependent on the used oxidation procedure. Usually, a C/O ratio from 1.8 to 2.5 is achieved. Since graphite is a very inert mineral, it can undergo oxidation only with very strong oxidation agents. Basically, two oxidizing agents are used to prepare graphite oxide: potassium permanganate [16] and potassium chlorate [17,18]. The use of potassium chlorates leads to the synthesis of GO with mainly hydroxyl and epoxy functionalities. On the other hand, the use of potassium permanganate provides GO with mostly carboxyl functionalities. In an optimized method (sometimes termed the Tour method) [19], graphite powder is oxidized in a mixture of sulfuric and phosphoric acid in the ratio 9:1. Phosphoric acid is crucial for stabilizing the reaction. This method was further modified by reducing the overall time of the synthesis to one sixth of the original procedure [20]. 

Graphite oxides and graphene oxides can be used as precursors for graphene synthesis or the synthesis of graphene derivatives such as hydrogenated and halogenated graphenes [21,22,23,24,25,26]. Graphite oxides can be used for the synthesis of graphene (by chemical or thermal reduction). It is a well-known fact that graphene has unique electrical, optical and mechanical properties [27,28,29].

Graphene oxide itself is a suitable material for the fabrication of membranes or foils [30]. The prepared graphite oxide can also undergo subsequent re-oxidation [31,32]. During the re-oxidation, the typical graphene structure disappears, forming an “amorphous” structure called graphene acid. Graphene acid and graphene oxides have great potential to remove heavy metals and other pollutants from water [33,34,35]. Heavy metals (Hg, Cd, As, Cr, Tl, Pb, Zn, etc.) are metallic chemical elements that are toxic or poisonous at low concentrations. Moreover, they are also non-degradable and persistent. Several methods have been explored to remove heavy metals from contaminated water, such as chemical precipitation, ion exchange, adsorption, reverse osmosis, solvent extraction and electrochemical treatment [36,37]. 

In this paper, the potential of ceramic foam filters was combined with graphene oxide in order to evaluate the possibility of using this composite material for the purification of polluted water from selected heavy metals in low concentrations.

## 2. Materials and Methods 

For the graphene oxide suspension (GO), pure graphite microparticles (2–15 μm, 99.9995%, from Alfa Aesar) were used. H_2_SO_4_ (98%), H_3_PO_4_ (85%), KMnO_4_ (99.5%), H_2_O_2_ (30%) and Ba(NO_3_)_2_ (99.5%) were obtained from Penta, Prague, Czech Republic. For sorption experiments, we used Pb(NO_3_)_2_, Cd(NO_3_)_2_ and Zn(NO_3_)_2_.6H_2_O from Lach-Ner, Neratovice, Czech Republic.

The carbon-bonded filters were prepared from fine aluminum oxide (Martoxid MR 70, Martinswerk, Bergheim, Germany, 99.80 wt.% Al_2_O_3_, *d*_90_ ≤ 3.0 μm) in combination with several carbon sources. As a binder, a modified coal tar pitch powder (Carbores P, Rütgers, Castrop-Rauxel, Germany, d_90_ < 0.2 mm) was selected. Furthermore, fine natural graphite (AF 96/97, Graphit Kropfmühl, Hauzenberg, Germany, 96.7 wt.% carbon, 99.8 wt.% <40 μm) and carbon black powder (Luvomaxx N-991, Lehmann & Voss & Co., Hamburg, Germany, carbon content ≥99.0 wt.%, primary particle size of 200–500 nm) were added in small amounts. For the dispersion in water and the stabilization of the water-based slurry, the following additives were introduced: ligninsulfonate (C12C, Otto-Dille, Norderstedt, Germany) as a wetting agent and temporary binder, modified polycarboxylate ether (Castament VP95L, BASF, Rhein, Germany) as a dispersing agent, and alkylpolyalkyleneglycolether (Contraspum K1012, Zschimmer & Schwarz, Lahnstein, Germany) as an antifoam agent.

Graphene oxide was analyzed by a broad spectrum of analytic methods. These methods are described in detail in the Supporting Information (SI). The morphology was investigated by scanning transmission electron microscopy (STEM, Tescan Lyra, Tescan, Brno, Czech Republic), the structure was analyzed by X-ray diffraction (XRD, Bruker D8 Discoverer, Bruker Bruker AXS GmbH, Karlsruhe, Germany) and Raman spectroscopy (InVia Raman microscope, Renishaw, Wotton-under-Edge, England). The chemical composition was studied by energy-dispersive X-ray spectroscopy (EDS, Oxford instruments, HighWycombe, UK), elemental analysis (EA, PE 2400 Series II CHNS/O Analyzer, Perkin Elmer, Waltham, USA) and X-ray photoelectron spectroscopy (XPS, ESCAProbeP spectrometer, Omicron Nanotechnology, Taunusstein, Ltd., Germany). Optical microscopy (OM, VHX-200 D, Keyence, Osaka, Japan) was used to study the surface of the ceramic foam filters. Atomic absorption spectroscopy (AAS, Agilent 280FS AA, Agilent Technologies, Santa Clara) was used for the determination of the concentration of zinc, cadmium and lead. 

The graphene oxide (GO) suspension was prepared according to the modified Tour’s method. This method is described in detail in the Supporting Information (SI). The final GO slurry contained approx. 5 g of graphene oxide and 250 mL of water, which is corresponding to the concentration of 20 g/L.

The carbon-bonded filters were produced via the Schwartzwalder process (also known as the replica technique), by using a two-step approach. As substrates, 100 × 100 × 22 mm^3^ polyurethane foams with an average porosity of 10 ppi (pores per inch) were selected. The foams were first impregnated by a thick water-based slurry and squeezed to remove the excess material. Afterwards, the same samples were cold spray-coated with a second, thinner slurry to achieve the desired wall thickness [38]. After drying, the filters were thermally treated in retorts filled with calcined petcoke (Müco, Essen, Germany, particle size between 0.2 and 2 mm), in order to prevent oxidation of the carbon fraction. The maximum temperature of 800 °C was reached with a heating rate of 1 °C/min, additional dwell steps of 30 min for every 100 °C, and a final holding time of 180 min at 800 °C.

A home-made dip-coating device was used for the coating of the ceramic foam filter using a graphene oxide suspension. The GO/water suspension with a concentration of 20 g/L was sonicated using an ultrasonic needle immediately before the dip-coating process. After this process, coated filters were placed into a vacuum dryer for 24 h at 30 °C at p/p^0^ = 0.3.

**Figure 1:**
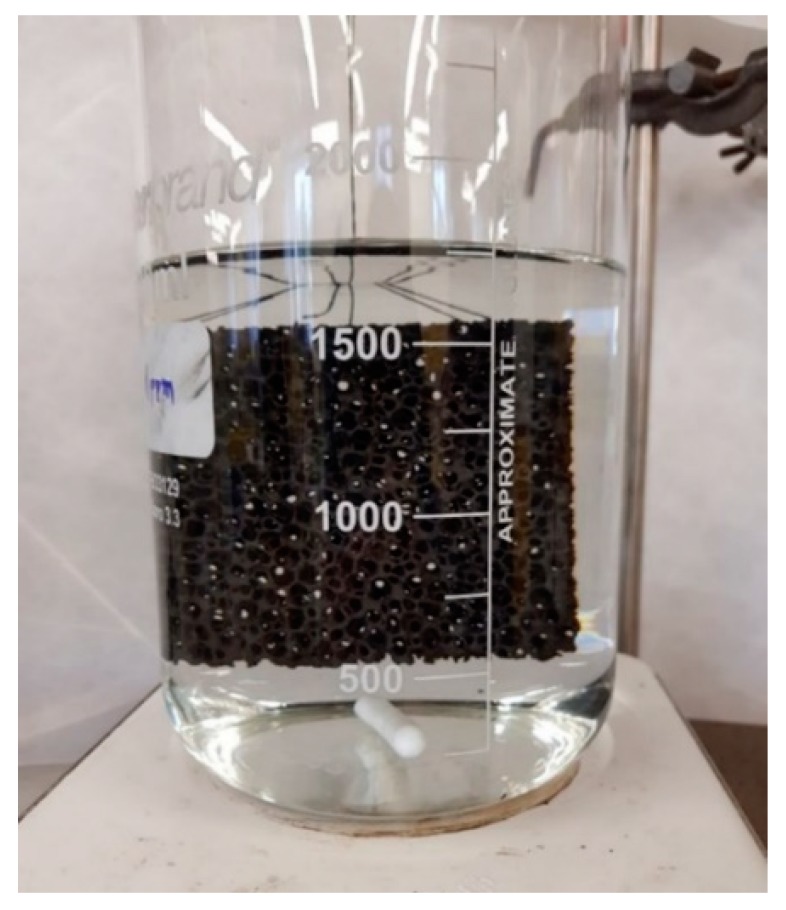
Sorption experiment of the coated ceramic foam filter.

The sorption experiment was performed in beaker with 1750 mL of solution containing 1 ppm of Zn^2+^, 1 ppm of Pb^2+^ and 1 ppm of Cd^2+^ (see Figure 1). The concentration of metals was verified using AAS. The solution was stirred using a magnetic stirring bar for 2 h. After the sorption, the filter was removed from the solution and the concentration of metals was measured again using AAS. 

Finally, the maximal sorption capacity of the prepared GO was tested using 1 g of GO and 1 L of solution, containing 5 wt.% of Zn^2+^, Pb^2+^ or Cd^2+^. In such concentrations, metals are in large surplus. The suspensions were stirred using a magnetic stirring bar for 2 h. After the sorption, GO was removed from the solution by centrifugation and further washed by distilled water. After drying, its chemical composition was measured using EDS. 

## 3. Results and Discussion 

Graphene oxide (GO) was synthesized using the modified Tour method. This sample was analyzed by TEM, EDS, EA, XPS, XRD, BET and Raman spectroscopy. GO was used for the nano-functionalization of carbon-bonded alumina-based ceramic foam filters. Coated filters were used for the purification of water contaminated by lead, zinc and cadmium ions. 

**Figure 2:**
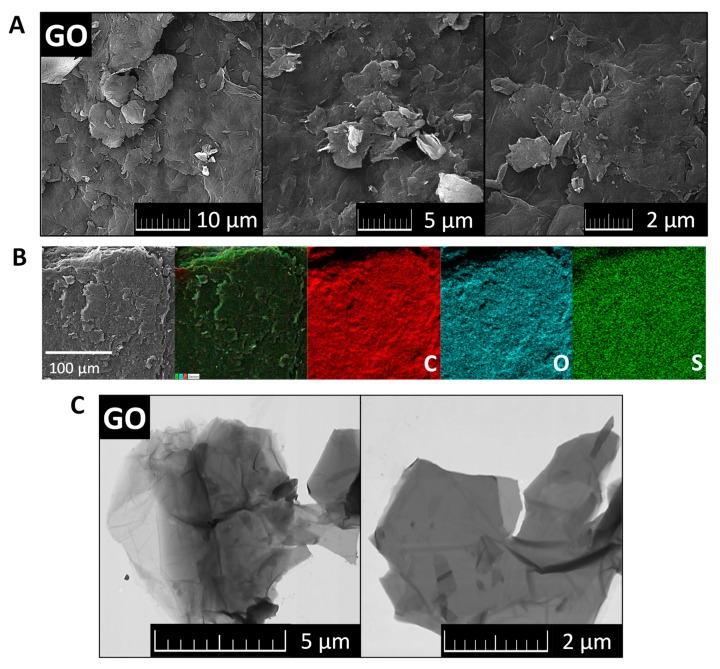
SEM micrographs (**A**), EDS maps of elements (**B**) and TEM images of GO (**C**).

**Figure 3:**
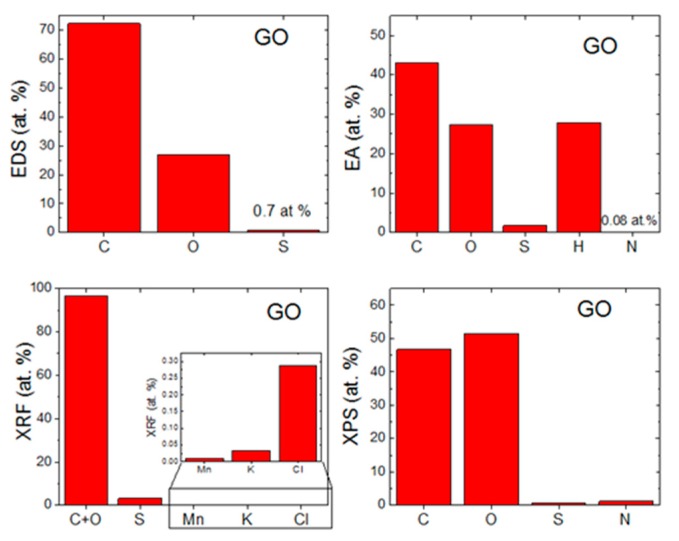
Comparison of chemical composition obtained by (**a**) EDS, (**b**) EA, (**c**) XRF and (**d**) XPS.

The morphology of GO was investigated by SEM and TEM (see Figure 2). SEM micrographs (Figure 2A) showed that GO had a high tendency to agglomerate and assemble layered structures in the form of foils. Such behavior is useful for further coating of the ceramic foam filters. TEM micrographs (Figure 2C) displayed the individual graphene oxide sheets. Their size is approx. 5 μm in diameter, which is in good agreement with the starting graphite powder. According to the TEM images, graphene oxides had less than 10 layers in a direction perpendicular to the sheets. EDS elemental maps in Figure 2B showed that individual elements were quite homogeneously distributed. Except for carbon and oxygen, sulfur was detected. This impurity was present due to the use of sulfuric acid during the graphite oxidation. According to EDS, the chemical composition was 72.3 at.% of carbon, 27.0 at.% of oxygen and 0.7 at.% of sulfur. The calculated C/O ratio was 2.7. The obtained composition is compared with other analytic methods in Figure 3. 

The chemical composition was also measured by EA (see Figure 3). From the principle of this method, only nonmetal elements can be detected. On the other hand, the hydrogen content can be measured, which is very useful for further analysis of individual oxygen and hydrogen-containing functionalities, such as hydroxyls, carbonyls and carboxyls. The chemical composition of GO was 43.0 at.% of carbon, 27.4 at.% of oxygen, 27.8 at.% of hydrogen, 1.8 at.% of sulfur and 0.1 at.% of nitrogen. From these data, the C/O ratio of 1.6 was obtained. Let us note that the oxygen content is determined indirectly in this technique, hence the C/O ratio differs from the results obtained by EDS. 

**Figure 4:**
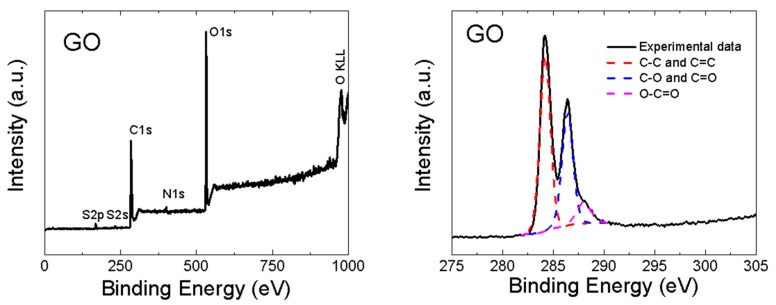
XPS spectra of GO: survey spectra (**left**) and C1s detail (**right**).

GO was also analyzed by XPS. In the XPS survey spectra (see the left panel in Figure 4), the C1s peak was visible at ∼284.4 eV and the O1s was found at ∼532.4 eV. According to XPS, the chemical composition of GO was 46.7 at.% of carbon, 51.3 at.% of oxygen, 0.7 at.% of sulfur and 1.3 at.% of nitrogen. From these data, the C/O ratio of 0.9 was obtained. Let us note that XPS is a highly surface-sensitive method and the GO surface contains mainly carboxyl functionalities, hence the C/O ratio is very low. A comparison with other analytic methods is shown in Figure 3. XPS was also used to quantify the number of oxygen-containing functional groups (see the right panel in Figure 4). The following carbon bonding states were identified in the graphite oxides or graphene oxides: C=C (284.4 eV), C–C (285.2 eV), C–O (286.2 eV), C=O (287.8 eV) and O–C=O (289.0 eV). For the deconvolution of the C1s peak, we decided to group C=C and C–C together as well as C–O and C=O. The results confirmed that GO is highly oxidized, where the content of various carbon bonding states was 54.4% of C=C and C–C, 36.9% C–O and C=O and 8.7% of O–C=O.

**Figure 5:**
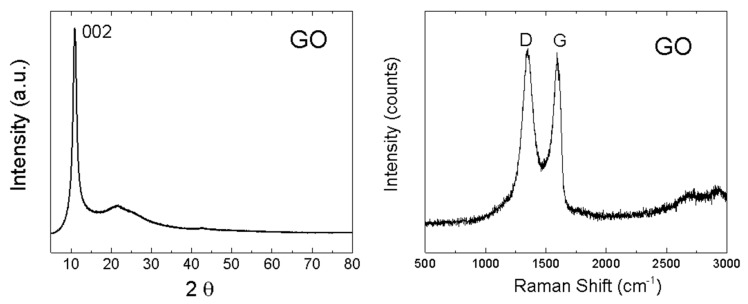
XRD diffraction pattern of GO (**left**) and Raman spectrum of GO (**right**).

The structure of GO was investigated by X-ray powder diffraction and Raman spectroscopy (Figure 5). While graphite exhibits the strongest reflection (002) at 26.5°, this reflection (002) was shifted to 10.9° for GO, indicating that the interlayer distance of GO increased to 8.1 Å, thus confirming a very high content of oxygen functionalities between individual layers. Using the Debye–Scherrer method, the average size of 57 Å in the 001 direction was calculated. This corresponds to 7 GO sheets in the 001 direction. In the Raman spectrum, two major bands are clearly visible [39,40]. The G-band was found at 1591 cm^−1^, representing sp^2^ bonded carbon atoms. Another band, termed D-band, was observed at 1348 cm^−1^ [41,42]. The D-band indicates defects in the graphene layer, which are mostly associated with sp^3^ hybridization of carbon atoms (mostly due to the presence of oxygen functionalities). To consider the level of oxidation of GO, the D/G ratio was calculated (intensities I_D_/I_G_). The obtained I_D_/I_G_ ratio was 1.02, suggesting a high concentration of oxygen functionalities [43]. 

**Figure 6:**
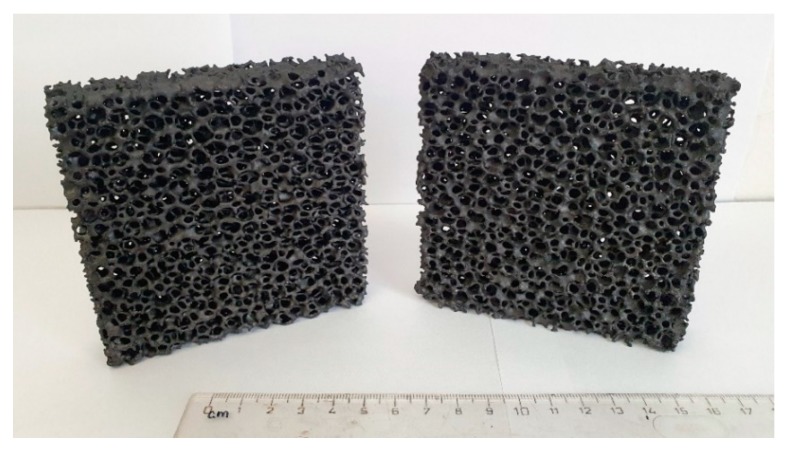
Photography of ceramic foam filters before the dip-coating (**left**) and after the dip-coating (**right**).

**Figure 7:**
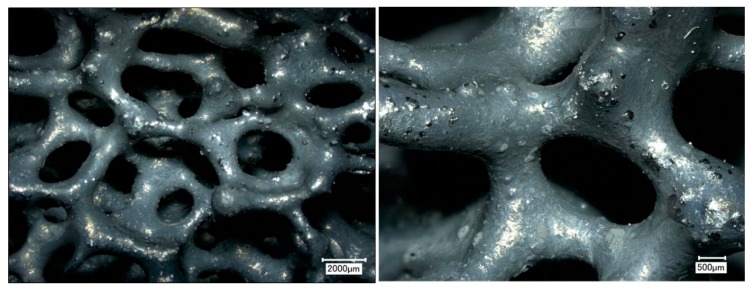
Optical microscopy of ceramic foam filter before the dip-coating: 20x magnification (**left**) and 50x magnification (**right**).

Ceramic foam filters were prepared by the Schwartzwalder process. Photographs of the filters before and after the coating are shown in Figure 6. No significant changes were observed. As can be seen from the photograph, the prepared filters do not possess the same surface area as granular activated carbon (GAC) or other high-surface-area sorbents. The main advantage of ceramic foam filters is their high permeability, which, in their prevalent application as melt filters in metallurgy processes, prevents the molten metal from freezing within the structure during operation. Optical microscopy was used to analyze the microstructure before the coating application (see Figure 7). According to the obtained micrographs, there are no visible defects, such as cracks or inhomogeneities. Such filters seem to be suitable for the coating using graphene oxide. Sufficient affinity of GO to Al_2_O_3_-C filters was previously confirmed [38]. 

After the coating, the efficiency of the sorption was analyzed by AAS. A comparison of the obtained concentrations is shown in Table 1. The concentration of cadmium and lead ions dropped below the detection limit of AAS, resulting in an efficiency of over 99.4% for cadmium sorption and over 95.0% for lead sorption. In the case of zinc ions, the efficiency of sorption was 99.2%. Such results showed very promising potential of the coated Al_2_O_3_-C filters for water treatment. In addition, after the experiment the solution was analyzed to check whether some GO was released during the sorption process. The solution was filtered using a Nylon filter membrane and no graphene oxide was collected, suggesting sufficient adhesion of GO to the substrate.

The maximal sorption capacity of GO was measured by EDS obtained as an average from five measurements. The obtained values were 35 mg of Cd^2+^/1 g of GO, 265 mg of Pb^2+^/1 g of GO and 11 mg Zn^2+^/1 g of GO, respectively. These results are also in good agreement with the data published for graphite oxides prepared by Hummers’ method [44]. 

## 4. Conclusions 

In this paper, graphene oxide was prepared and characterized. Graphene oxide with a high number of oxygen functionalities was obtained. In the next step, graphene was used for the coating of carbon-bonded ceramic foam filters that were prepared by the Schwartzwalder process. The coated filters were then used in a sorption experiment, where the uptake efficiency for lead, cadmium and zinc ions was tested. The results of the research are very promising, since the efficiency was very high. The concentrations of metals dropped from 1 ppm to several ppb in all cases. Our composite filters can thus be used for water treatment where the concentration of waste metals is not very high, otherwise the maximum capacity of the filter would soon be reached. Similar materials could be used in building applications for self-cleaning surfaces. Another potential application of such composites is in the steel industry, where the modification of the ceramic foam surface can influence microroughness and, therefore, also the efficiency of metal melt filtration. 

## Figures and Tables

**Table 1:** Concentration of metals before and after the sorption.

	Cd^2+^ (ppm)	Pb^2+^ (ppm)	Zn^2+^ (ppm)
Before sorption	1.0	1.0	1.0
After sorption	<0.006	<0.05	0.008
Efficiency of sorption	>99.4%	>95.0%	99.2%

## Supplementary Materials

The supplementary material is available online at https://www.mdpi.com/1996-1944/13/8/2006/s1.
