# Supplementary material for: Carbon-Bonded Alumina Filters Coated by Graphene Oxide for Water Treatment
Source: Materials (Basel). 2020 Apr 24;13(8):2006. doi: 10.3390/ma13082006 (PMC7215612; doi:10.3390/ma13082006)
Supplement: Supplementary file 1 [file materials-13-02006-s001.pdf]

materials

MDPI

Supplementary Materials

Carbon-Bonded Alumina Filters Coated by Graphene Oxide for Water Treatment

Ondřej Jankovský [1,*], Michal Lojka [1], Adéla Jiříčková [1], Christos G. Aneziris [2], Enrico Storti [2] and David Sedmidubský [1]

[1] Department of Inorganic Chemistry, Faculty of Chemical Technology, University of Chemistry and Technology, Technická 5, 166 28 Prague 6, Czech Republic; michal.lojka@vscht.cz (M.L.); Adela.Jirickova@vscht.cz (A.J.); sedmidub@vscht.cz (D.S.)

[2] Technische Universität Bergakademie Freiberg, Institute of Ceramic, Glass and Construction Materials, Agricolastraße 17, 09599 Freiberg, Germany; aneziris@ikgb.tu-freiberg.de (C.G.A.); enrico.storti@ikgb.tu-freiberg.de (E.S.)

* Correspondence: ondrej.jankovsky@vscht.cz; Tel.: +420-220-44-2002

Received: 29 March 2020; Accepted: 20 April 2020; Published: date

Experimental Procedures

The graphene oxide (GO) suspension was prepared according to the modified Tour's method. A mixture of concentrated sulfuric acid and phosphoric acid in a volume ratio 9:1 (360:40 ml) was cooled to 5 °C. Next, graphite (3.0 g), and subsequently potassium permanganate (18.0 g), was added. The ongoing reaction heated the mixture itself to ≈20 °C due to the exothermic process. The reaction mixture was stirred and then heated to 50 °C for 1 h. Then, the mixture was cooled to 20 °C and poured onto 500 g of ice. After the ice dissolved, 30% hydrogen peroxide was added (50 ml) to remove the remaining unreacted potassium permanganate and manganese dioxide. The obtained GO was then purified by repeated centrifugation and redispersing in deionized water until a negative reaction on sulfate ions with Ba(NO3)2 was achieved. After the centrifugation, the final GO slurry contained ≈5 g of graphene oxide and 250 ml of water, which corresponds to the concentration of 20 g/L.

Methods

The morphology was investigated using scanning electron microscopy (SEM) with an field emission gun (FEG) electron source (Tescan Lyra dual beam microscope). Elemental composition and mapping were performed using an energy-dispersive spectroscopy (EDS) analyzer (X-MaxN) with a 20 mm^2 SDD detector (Oxford instruments, High Wycombe, UK) and AZtecEnergy software (Oxford instruments, HighWycombe, UK). To conduct the measurements, the samples were placed on a carbon conductive tape. SEM and SEM-EDS measurements were carried out using a 10 kV electron beam.

X-ray fluorescence (XRF) analysis was performed by an ARL PERFORM'X sequential WD-XRF spectrometer (Thermo Scientific, Waltham, MA, USA) equipped with an Rh anode end-window X-ray tube type 4 GN, fitted with a 50 μm Be window. All peak intensity data were collected by the software Oxsas (Thermo Scientific, Waltham, MA, USA) in a vacuum. The generator settings-collimator-crystal-detector combinations were optimized for all 82 measured elements, with an analysis time of 6 s per element. The obtained data were evaluated by the standardless software Uniquant 5 integrated in Oxsas. The analyzed powders were pressed into pellets about 5 mm thick with a diameter of 40 mm without any binding agent and covered with 4 μm supporting polypropylene (PP) film.

The scanning transmission electron microscopy (STEM) was performed with a Tescan Lyra dual-beam microscope equipped with an FEG electron source and STEM sample holder. To conduct the

measurements, the sample suspension was drop casted on a 200 mesh Cu TEM grid and dried in a vacuum oven (50 °C). STEM measurements were carried out using a 30 kV electron beam.

X-ray powder diffraction (XRD) was carried out at room temperature on a Bruker D8 Discoverer (Bruker Bruker AXS GmbH, Karlsruhe, Germany) 45 powder diffractometer with parafocussing Bragg–Brentano geometry by using CuKα radiation 46 (λ = 0.15418 nm, U = 40 kV, I = 40 mA). Data were scanned over the angular range 5°–80° (2θ). Interlayer 47 distances and particle sizes were calculated in PANalytical's X'Pert High Score software (version 4.0, Almelo, Netherlands).

Combustible elemental analysis (EA) was performed using a PE 2400 Series II CHNS/O Analyzer (Perkin Elmer, Waltham, MA, USA). The instrument was used in CHN operating mode to convert the sample elements to simple gases (CO_2, H_2O and N_2). The PE 2400 Analyzer automatically performed combustion, reduction, homogenization of product gases, separation and detection. An MX5 microbalance (Mettler Toledo, Columbus, OH, USA) was used for precise weighing of the samples (1.5–2.5 mg per sample). The internal calibration was performed with N-phenyl urea.

Raman spectroscopy was performed with an InVia Raman microscope (Renishaw, Wotton-under-Edge, England) in backscattering geometry with a CCD detector). A DPSS laser (532 nm, 50 mW) with the applied power of 5 mW and a 50× magnification objective was used for the measurement. Instrument calibration was achieved with a silicon reference which gives a peak position at 520 cm^{-1} and a resolution of less than 1 cm^{-1}. The samples were suspended in deionized water (1 mg/ml) and ultrasonicated for 10 minutes. The suspension was deposited on a small piece of silicon wafer and dried.

High-resolution X-ray photoelectron spectroscopy (XPS) was performed with an ESCAProbeP spectrometer (Omicron Nanotechnology Ltd., Taunusstein, Germany) with a monochromatic aluminum X-ray radiation source (1486.7 eV). The sample was applied on a conductive carbon tape before the analysis. Wide-scan surveys of all elements were performed, with subsequent high-resolution scans of the C 1s and O 1s.

Optical microscopy (OM) was performed with the aid of a digital light microscope VHX-200 D (Keyence, Osaka, Japan). The uncoated carbon-bonded filters were investigated by using the sidelight source. The objective allows for magnifications in the range of 20×–200×.

The concentrations of metal ions before and after the sorption experiment were determined by means of atomic absorption spectroscopy (AAS, Agilent Technologies, Santa Clara, CA, USA) on an Agilent 280FS AA device using a flame-atomization technique. Measurements were carried out in acetylene-air flame.

© 2020 by the authors. Submitted for possible open access publication under the terms and conditions of the Creative Commons Attribution (CC BY) license (http://creativecommons.org/licenses/by/4.0/).
